# Milk Composition during Lactation Suggests a Mechanism for Male Biased Allocation of Maternal Resources in the Tammar Wallaby (*Macropus eugenii*)

**DOI:** 10.1371/journal.pone.0051099

**Published:** 2012-11-30

**Authors:** Kylie A. Robert, Shannon Braun

**Affiliations:** Department of Zoology, La Trobe University, Bundoora, Australia; University of Manitoba, Canada

## Abstract

Recent research has found empirical evidence in support of the Trivers-Willard Hypothesis that offspring sex allocation is correlated with maternal investment. Tammar wallabies birthing sons have higher investment ability; however a mechanism for sex specific differential allocation of maternal resources in wallabies remains elusive. In metatherians the majority of maternal investment is during lactation. To examine if differential allocation occurs during lactation, we measured total milk protein, lipid and carbohydrates, from mothers with male and female pouch young, during phase 2B (100–215 days post partum) and phase 3 (215–360 days post partum) of lactation. Mothers of sons allocated significantly higher levels of protein than mothers of daughters during phase 2B of lactation, however no sex specific difference in maternal allocation was found for lipids, carbohydrates, or any milk component during phase 3 of lactation. We were unable to measure milk production to establish any differences in the amount of milk allocated. However, with the production of more milk comes a dilution effect on milk components. Given that we find no apparent dilution of milk components may suggest equality in milk production. Offspring body weight at 14 months of age was related to protein allocation during phase 2B of lactation, providing a maternal mechanism for differential allocation with fitness consequences. We believe collection of earlier phase 2A (0–100 days post partum) milk may yield important results given that differential investment in metatherians may be most apparent early in lactation, prior to any significant maternal investment, when a decision on termination of investment can be made with very little energetic loss to the mother. Interestingly, small mothers did not birth sons and better maternal condition was associated with raising sons. These data are in support of TWH and demonstrate a potential mechanism through which condition dependent and sex specific maternal investment may occur.

## Introduction

Despite the voluminous literature investigating sex-allocation theory a general consensus for biased sex ratios in mammals has not yet emerged [1], [2]. This is in part due to how few empirical studies test the underlying assumptions of hypotheses [3]. Hypotheses on sex allocation predict that maternal investment should be biased towards the sex that gains more reproductive value from higher maternal investment [4]. Under the Trivers-Willard Hypothesis (TWH) mothers should invest in the sex that, under their current condition, will maximise their reproductive potential [5]. Mothers with greater investment potential are more able to produce better conditioned offspring (for example, larger, stronger and fitter offspring) than those in poorer condition. In polygynous species matings are monopolized by a few larger males and hence sire many more offspring per season than their female counterparts. Under such conditions male reproductive success is dependent on body condition, which in many cases is dependent on maternal investment potential during early development (Reviewed in [6]). Therefore, in polygynous species, mothers in good condition should invest in sons, that will sire many more grand-offspring than daughters. While females in poor condition should invest in daughters who are much more likely to reproduce than poor conditioned sons [5]. Sex biased offspring investment is often realised in either two ways that are not mutually exclusive, 1) a greater probability of producing one sex over the other (or in the case of litters or clutches, sex ratios biased towards one sex) [5], [7]; or 2) a greater care or provisioning towards one sex [3], [8].

Mammals are a particularly good taxon for the study of maternally derived sex biased provisioning, as maternal post-natal investment is dependent primarily on the milk produced by the mother [9]. Although milk production scales with maternal weight [10], and in some mammals so does composition [10], mothers can increase the amount of milk in an effort to increase offspring growth or survival [11]. Within milk components, protein is the component most directly related to offspring growth [9], [12]. According to the predictions of the TWH, sex biases in milk production and composition should be found among mammals. Despite this belief few studies have examined differential provisioning to male and female offspring through milk production or composition. The first study to report such a bias, found a greater amount of milk, as well as greater percent of milk protein was provided to sons over daughters in deer [13], although the biases found in mineral composition are not so clearly supportive of TWH [14]. Since this first study, further studies in primates [15], [16], show primiparous mothers produce milk with more protein and lipids in favour of sons.

Sex-allocation theory is poorly understood among Australia’s metatherian taxa [17], which in the past have suffered greatly from the extensive loss of biodiversity since European settlement [18]. Unbalanced sex ratios frequently occur in marsupials [17], [19] and male biased sex ratios are increasingly problematical in captive breeding programs that play an important role in conservation and management [20]. It is therefore essential that a greater understanding of facultative sex allocation in marsupials be developed. Robert *et al* [21] conducted the first study to empirically test for differences in the investment potential of mothers birthing sons versus daughters by cross fostering wallaby pouch young between mothers. Mothers who birthed son’s showed significantly higher weaning success than those birthing daughters regardless of the sex fostered. These findings were consistent with those of Sunnucks and Taylor [22] where maternal mass was positively correlated with the probability of a male offspring in the pouch and fulfils the prediction of TWH whereby mothers of greater investment capacity bias offspring sex in favour of sons. Male offspring incur greater energy costs to mammalian mothers [23], however the mechanisms by which sex dependent resource allocation occurs in wallabies is not yet understood.

The tammar wallaby (*Macropus eugenii*) is the most widely studied macropod species, and one of only two macropods with strictly seasonal breeding and highly synchronised births regulated by changes in day length [24]. This highly seasonal breeding ensures that peak lactation coincides with predictable winter rainfall. Most young are born in mid-summer from late January to early February (in the Southern hemisphere) and females experience a post-partum oestrus. The embryo conceived post-partum develops to the blastocyst stage then remains dormant during lactation of the current pouch young. Blastocyst re-activation occurs through the loss of the current young and removal of the sucking stimulus cues, but only prior to the winter solstice in June (Southern hemisphere), after this period re-activation cannot occur until after the summer solstice in late December in response to decreasing day length [25]. This ability to re-activate a stored blastocyst before the winter solstice allows for early termination of a females current investment early in the season and reinvestment in a new pouch young with minimal cost to the mother. A great deal is known about the composition of milk and development of pouch young in tammar wallabies [26], [27]. The composition of milk changes dramatically throughout lactation with four phases recognised (phase 1: pre partum ∼26 day gestation, phase 2A: 0–100 days post partum, phase 2B: 100–215 days post partum and phase 3: 215–360 days post partum). The end of phase 2B is considered a transitional phase that is characterised by a decrease in carbohydrates and an increase in lipids and proteins [27]. In the tammar wallaby the lactating female regulates both the rate of milk production and the composition of the milk and this then determines the rate of pouch young growth and development, irrespective of the age of the pouch young [27]. Tammars provide an opportunity to link maternal investment ability [21] with a mechanism of investment, through the study of sex specific, differential allocation of maternal resources post-natally during lactation. Here we directly test sex specific differences in protein, lipid and carbohydrate milk components across phase 2B and phase 3 of lactation. Significant male biased differences will provide further support for a TWH prediction that mothers invest more heavily in male offspring through post-natal investment.

**Figure 1:**
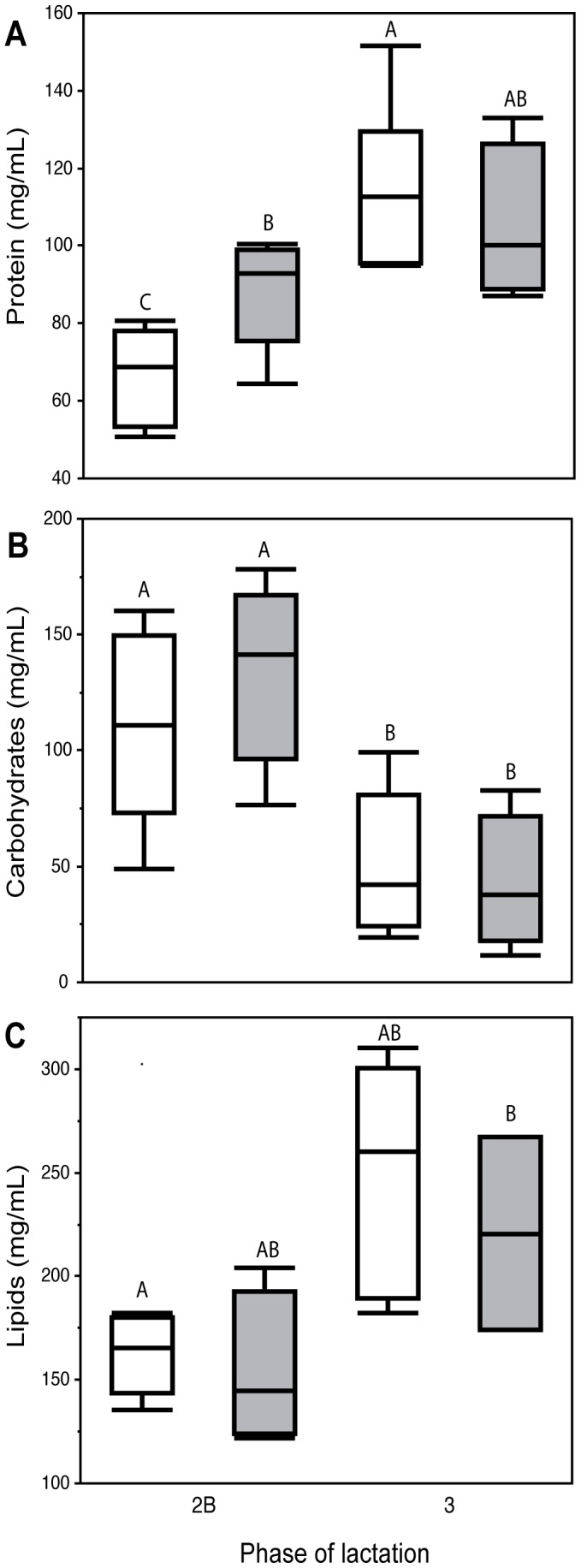
Milk components by offspring sex. (a) protein (b) carbohydrate and (c) lipid content in milk during phase 2B and phase 3 of lactation in wallabies birthing sons (shaded) or daughters (unshaded). Females allocate significantly more protein to sons during Phase 2B of lactation than to daughters, while females allocate both carbohydrates and lipids equally to sons and daughters during phase 2B and 3 of lactation. Levels not connected by the same letter are significantly different (*post-hoc* Student’s t-test).

**Figure 2:**
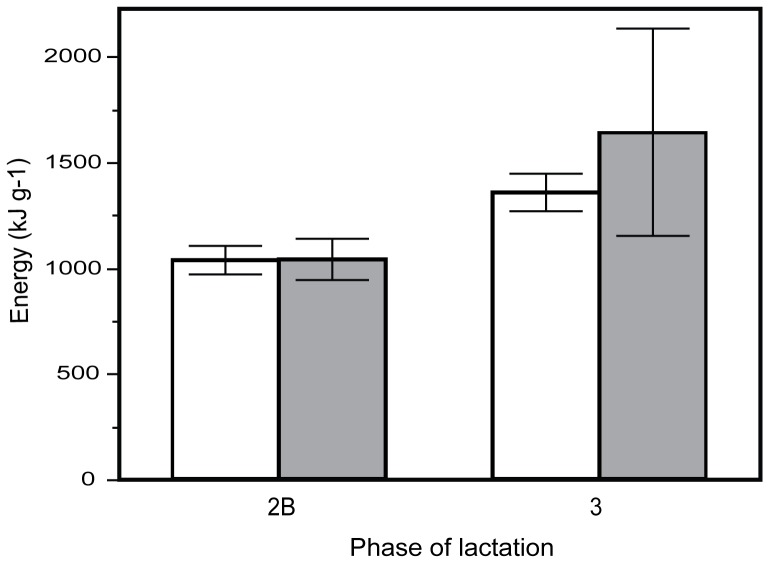
Gross milk energy content during phase 2B and phase 3 of lactation. Mean changes (± s.e.) in the energy content of milk from Tammar wallaby mothers raising sons (shaded bars) and those raising daughters (open bars) during phase 2B and phase 3 of lactation. Despite the allocation of different milk components the total energy content of the milk is not significantly different between the milk allocated to sons and daughters.

## Materials and Methods

Ethics statement: All animal work was conducted according to relevant national and international guidelines. The project was approved by La Trobe University Animal Ethics Committee (AEC11-05) and Department of Sustainability and Environment Research Permit (DSE 10005722).

**Figure 3:**
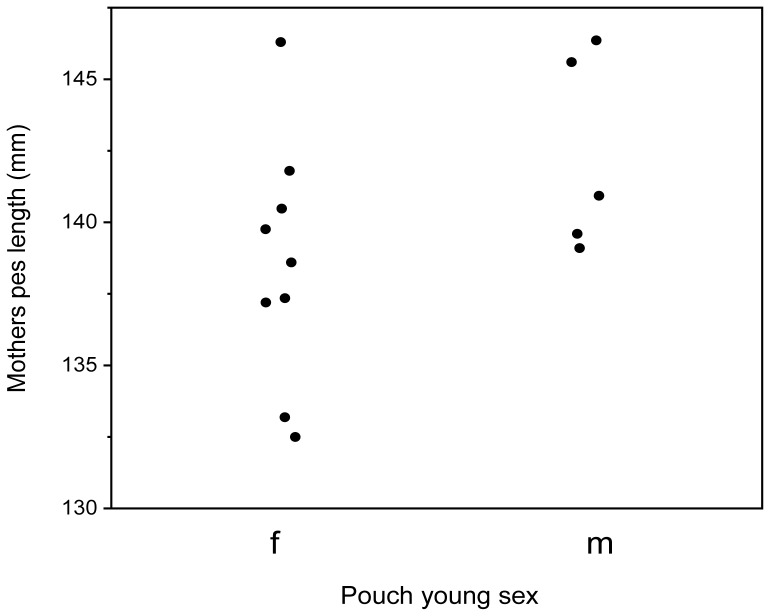
Smaller females only produce daughters. Maternal body size (pes length) and offspring sex. Females with a pes length of less than 139 mm only produced daughters. Although, mothers pes length did not predict offspring sex (Logistic regression: ChiSq = 2.08, *p* = 0.151).

**Figure 4:**
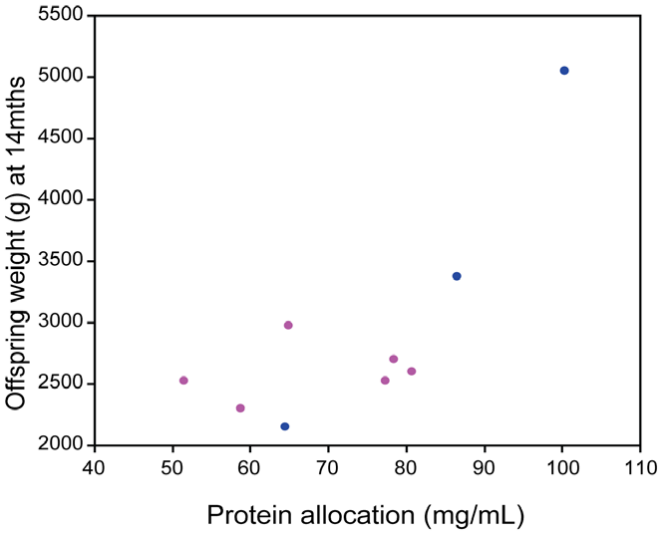
Protein allocation during phase 2B of lactation influences offspring weight into adulthood. Maternally allocated proteins during phase 2B of lactation influences offspring body weight at fourteen months of age.

The tammar wallabies (*Macropus eugenii derbianus*) originated from wild caught individuals from Tutanning Nature Reserve, Western Australia (32°33’ S, 117°20’ E) and are part of a captive breeding colony housed at La Trobe University, Australia. Wallabies are housed in naturally vegetated outdoor enclosures, supplemented with *ad libitium* Kangaroo cubes (Glen Forrest Stockfeeders, Western Australia), mixed fruit, vegetables, and water.

Milk samples were collected from lactating females during two sampling periods in July and October 2011. All females with the exception of two individuals were two years of age and reproducing for the first time. Fifteen females were carrying pouch young in July (9 with daughters, 6 with sons), mean age of female pouch young was 130 days (range 110–146) and male pouch young was 136 days (range 107–159). In October, eleven females were carrying pouch young (7 with daughters, 4 with sons), mean age of female pouch young was 235 days (range 217–255) and male pouch young was 232 days (range 218–250). Age was estimated based on growth tables and on the assessment of major developmental features (e.g. appearance of whiskers, eyes opening, pigmentation and growth stage of fur) to back calculate birth dates [27]–[29]. Four pouch young were lost between sampling periods and abandonment of a breeding effort during lactation is an evolutionary adaptation in macropods with 45–65% pouch young loss expected in Tammar wallabies [30], [31].

**Table 1:** Separate analysis of variance for effect of maternal traits on milk composition in phase 2B and phase 3 of lactation in the Tammar wallaby.

		Milk components					
		Protein		Carbohydrates		Lipids	
Phase of Lactation	Maternal factors	F	*P*-value	F	*P*-value	F	*P*-value
2B	Body weight	3.063	0.118	0.604	0.459	0.064	0.806
	Body size	2.936	0.125	0.329	0.582	0.000	0.995
	Body mass index	3.036	0.120	0.641	0.446	0.077	0.788
	Change in body weight	0.506	0.497	1.012	0.344	0.887	0.374
3	Body weight	1.466	0.293	0.568	0.493	0.003	0.957
	Body size	1.382	0.305	0.544	0.502	0.004	0.952
	Body mass index	1.476	0.291	0.530	0.507	0.002	0.962
	Change in body weight	0.427	0.549	**9.241**	0.038*	0.139	0.728

Maternal factors had no influence on milk components during early lactation (phase 2B), while change in maternal weight during late lactation influenced milk carbohydrates.

At the time of capture females were placed within hessian sacks, weighed (g), pes (foot) length measured (mm) and sedated 5 minutes prior to milk collection with a mixture of Ketamine (10 mg/kg) and Xylazine (1.25 mg/kg) injected intramuscularly. To encourage milk let down females were also administered 0.2 IU Oxytocin (Illium Syntocin®) intramuscularly [27]. Pouch young were temporarily removed from the pouch (max. of 20 min), placed within cloth bags, weighed (g), head and pes length measured (mm), sexed, microchipped, and then placed within a incubator (23°C, 95–100% humidity) for the period of separation [32]. All miilk that could be collected was manually expressed and collected in either microhaematocrit tubes or 1.5 mL eppendorf tubes by gently massaging the mammary gland and teat. Samples were then stored at −80°C until analysis. Furless pouch young were re-attached to the teat [32] and furred young were placed back into the pouch to re-attach themselves. Mother’s were kept within hessian sacks until fully recovered from sedation (∼ 1 hr) and checked prior to release to ensure pouch young had re-attached to the teat.

Milk samples were assayed for total proteins (Thermo Scientific - Micro BCA Protein Assay Kit, Product # 23235), lipids as described by Atwood & Hartman [33] and carbohydrates as described by Messer *et al.* [34]. The gross energy content of milk was estimated from the concentration of milk components, assuming the gross energy values of 24.6 kJ g^−1^ (protein), 16.1 kJ g^−1^ (carbohydrate), and 38.1 kJ g^−1^ (lipid) [8]. Milk components were statistically analysed by pouch young sex, phase of lactation and the interaction between sex and phase of lactation using mixed model effects with female identification entered as a random effect to avoid pseudo-replication. The influence of maternal traits on milk composition in both phases of lactation were analysed using separate analysis of variance (ANOVA). Maternal body condition was measured as a body mass index (BMI), calculated as the residuals of a linear regression of body mass vs. pes length (see [35] for detailed description of BMI calculation). The influence of maternal size (pes length) and body condition (BMI) on offspring sex was analysed by logistic regression. All statistical analysis was performed in JMP 8 (SAS institute).

## Results

As predicted and typical for macropod milk components there was a significant increase in both protein and lipids and a decrease in carbohydrates from phase 2B to phase 3 milk (protein: *F* = 26.42, *p*<0.001; lipids: *F* = 10.89, *p* = 0.007; carbohydrates: *F* = 15.03, *p* = 0.002; Fig. 1). A significant interaction effect was found between pouch young sex and phase of lactation with male pouch young receiving significantly more protein than females during phase 2B of lactation (pouch young sex * phase of lactation: *F* = 5.00, *p* = 0.045; Fig. 1). There was no significant interaction effect found for lipids or carbohydrates. Variation in milk composition was not explained by maternal body size, body weight, body mass index, or weight change during phase 2B of lactation, although carbohydrates were positively associated with maternal weight gain during phase 3 of lactation (Table 1). Despite differences in milk components the gross energy content of the milk did not differ between mothers raising different sexed offspring or across the two phases of lactation (pouch young sex * phase of lactation: *F* = 0.557, *p* = 0.470; Fig. 2). Interestingly, mothers of a small body size (pes length less than 139 mm) only produced daughters, although pes length did not predict offspring sex (Logistic regression: ChiSq = 2.08, *p* = 0.151) (Fig. 3) and maternal condition (BMI) was associated with better condition mothers raising sons (Logistic regression: ChiSq = 5.09, *p* = 0.024). Protein allocation during phase 2B of lactation has lasting fitness consequences with higher protein allocation significantly related to offspring body weight at fourteen months of age (ANOVA: *F* = 10.45, *p* = 0.014; Fig. 4).

## Discussion

In the current study we find evidence for the differential allocation of post-natal maternal resources, in favour of males, through higher protein content allocated during phase 2B of lactation. Allocation of higher protein content is suggestive of a bias in maternal provisioning towards sons; although we were unable to measure milk production or milk yield to establish if changes in composition are associated with the amount of milk produced [16]. Measuring milk production (particularly in early lactation phases) is technically more difficult in marsupials due to the pouch young’s highly altricial state, permanent attachment to the teat and limited ability to remain detached from the teat for the required length of time to collect sufficient samples. Hence it could be considered that marsupials in effect replace the umbilical cord for the teat during early development. However, in saying this it was equally difficult to collect milk from mothers raising male and female pouch young during the early phases of lactation and the volumes collected were equivalent so it does not appear that either sex receives more or less than the other. Despite the inability to measure milk production higher protein allocation during early lactation has important consequences on offspring body weight into adulthood with those males provisioned with the highest protein content milk early in development gaining the largest body sizes. These data are consistent with studies on Red deer [13], [14], Rhesus Macaques [15], [16], humans [36], [37] and the Trivers-Willard Model [5] that provides support for a mechanism by which wallabies with greater investment ability can allocate resources in favour of sons [21]. Lactation quantity and composition in macropods follows an endogenous maternal program independent of the pouch young sucking stimulus [26], [27] or the size of current pouch young when cross-fostered [38]. Therefore investment ability must depend on a pre-partum measure of condition [21]. Green *et al.* [26] found that the conversion of protein and milk energy to body materials in tammar wallabies is intermediate compared with other mammals, suggesting that slow growth rates in the early stages of development may be due to restricted milk production by mothers. Mothers investing in sons are generally in a condition when the lactation program is set, that is indicative of high investment capacity. Mothers in good body condition are therefore less likely to abandon the pouch young and thus can afford to provide more resources than mothers of poorer condition, during the early stages of development.

Higher levels of protein during phase 2B of lactation may be advantageous to males for multiple reasons. Primarily protein has direct relevance to growth in mammals [9], [14] and increased pre and post-natal consumption of protein may influence the development of adiposity in later life [39]. Tammar wallabies are polygynous and matings are highly competitive, with larger males being most successful [40]. Therefore the sons of mothers investing in protein rich milk are at a fitness advantage when this infers larger size in later life. It is quite clear that the transition phase (end of phase 2B) is also a time of essential immune transfer that prepares pouch young for increased pathogen exposure [41]. Therefore males receiving higher levels of protein may be receiving immunological benefits as well as advantages in growth and size [42].

In Rhesus Macaques (*Macaca mulatta*), primiparous mothers of sons produced milk richer in protein and lipid than primiparous mothers of daughters who had higher levels of carbohydrates [15], [16]. First born sons exhibited higher post-natal mortality, suggesting that primiparous mothers are more limited in their capacity to sustain the higher costs of lactation required for raising sons. In our study smaller mothers who had not completed their own growth could not produce sons (Fig. 3). This is consistent with life history theory where younger mothers face tradeoffs between reproduction and their own growth [43], [44].

We did not measure milk production so we cannot establish if there is a compensation effect between content of milk or milk yield as shown in Red Deer [13], [14] and Rhesus Macaques [45]. Estimated gross milk energy is not different between the sexes and what is surprising, is that the gross milk energy is not different between the two phases of lactation. Gross milk energy in macropods, including measures in the tammar wallaby have been shown to increase significantly from mid lactation to late lactation [26], [46] with measures taken later in lactation (∼360 days) when the proportion of lipids are at their highest. Our samples for phase 3 lactation were collected (∼220 days) prior to this period when lipids increase substantially which could explain why gross energy was not significantly different between the phases.

Our benign captive environment results in females of similar condition and nutrient access, that results in less variability in potential energy allocation throughout lactation. The study of wild individuals that show high variability in available resources [35] will be a profitable avenue of future investigation along with the development of techniques that will allow the measurement of milk production in early phases of lactation, however these may be very difficult to perform. Milk composition in another macropod, the Tasmanian pademelon (*Thylogale billardierii*) shows large differences in captive and wild populations that vary in nutritional intake [46]. Maternal diet is known to influence the composition of milk [47] and reduced food intake or suboptimal nutrient intake in will often result in altered milk composition and a reduced ability to raise young [48]–[50].

We intend to repeat this study with a larger group and additionally sample earlier phase 2A milk. During this early phase of lactation much of the equivalent developmental changes in eutherians occurs *in utero* [25]. For this reason the very early phase lactation in marsupials may show a unique composition of components unique to supporting the developing pouch young. For example, the recent discovery of unique anti-microbial peptides in tammar wallaby milk [51].This early phase 2A lactation has high variability in both whole milk proteins and whey proteins [52] and we believe that differential maternal investment (in particular with proteins) will be most evident in early phases of lactation, prior to any significant maternal investment. Maternal energetic investment into offspring during this time (phase 2A lactation) is negligible [26], [53], as offspring weight less than 20 grams, which is <0.5% of maternal body mass. With such negligible investment a decision on termination can be made with very little energetic loss to the female through disposal of the current young and re-activation of the stored blastocyst. Future studies into sex dependent, differential allocation of post-natal resources should consider the maternal cost of milk components, thereby linking differential investment with maternal cost and finally with maternal investment capacity.
